# Impairment of fragile X mental retardation protein-metabotropic glutamate receptor 5 signaling and its downstream cognates ras-related C3 botulinum toxin substrate 1, amyloid beta A4 precursor protein, striatal-enriched protein tyrosine phosphatase, and homer 1, in autism: a postmortem study in cerebellar vermis and superior frontal cortex

**DOI:** 10.1186/2040-2392-4-21

**Published:** 2013-06-26

**Authors:** S Hossein Fatemi, Timothy D Folsom, Rachel E Kneeland, Mahtab K Yousefi, Stephanie B Liesch, Paul D Thuras

**Affiliations:** 1 Department of Psychiatry, Division of Neuroscience Research, University of Minnesota Medical School, 420 Delaware St SE, MMC 392, Minneapolis, MN 55455, USA; 2 Department of Pharmacology, University of Minnesota Medical School, 310 Delaware St SE, MMC 392, Minneapolis, MN 55455, USA; 3 Department of Neuroscience, University of Minnesota Medical School, 321 Delaware St SE, MMC 392, Minneapolis, MN 55455, USA; 4 Department of Psychiatry, VA Medical Center, 1 Veterans Drive, Minneapolis, MN 55417-2399, USA

**Keywords:** Autism, RAC1, Homer 1, APP, STEP, BA9, Cerebellar vermis, Children, Adults

## Abstract

**Background:**

Candidate genes associated with idiopathic forms of autism overlap with other disorders including fragile X syndrome. Our laboratory has previously shown reduction in fragile X mental retardation protein (FMRP) and increase in metabotropic glutamate receptor 5 (mGluR5) in cerebellar vermis and superior frontal cortex (BA9) of individuals with autism.

**Methods:**

In the current study we have investigated expression of four targets of FMRP and mGluR5 signaling - homer 1, amyloid beta A4 precursor protein (APP), ras-related C3 botulinum toxin substrate 1 (RAC1), and striatal-enriched protein tyrosine phosphatase (STEP) - in the cerebellar vermis and superior frontal cortex (BA9) via SDS-PAGE and western blotting. Data were analyzed based on stratification with respect to age (children and adolescents vs. adults), anatomic region of the brain (BA9 vs. cerebellar vermis), and impact of medications (children and adolescents on medications (n = 4) vs. total children and adolescents (n = 12); adults on medications (n = 6) vs. total adults (n = 12)).

**Results:**

There were significant increases in RAC1, APP 120 kDa and APP 80 kDa proteins in BA9 of children with autism vs. healthy controls. None of the same proteins were significantly affected in cerebellar vermis of children with autism. In BA9 of adults with autism there were significant increases in RAC1 and STEP 46 kDa and a significant decrease in homer 1 vs. controls. In the vermis of adult subjects with autism, RAC1 was significantly increased while APP 120, STEP 66 kDa, STEP 27 kDa, and homer 1 were significantly decreased when compared with healthy controls. No changes were observed in vermis of children with autism. There was a significant effect of anticonvulsant use on STEP 46 kDa/β-actin and a potential effect on homer 1/NSE, in BA9 of adults with autism. However, no other significant confound effects were observed in this study.

**Conclusions:**

Our findings provide further evidence of abnormalities in FMRP and mGluR5 signaling partners in brains of individuals with autism and open the door to potential targeted treatments which could help ameliorate the symptoms of autism.

## Background

Autism is a neurodevelopmental disorder that is characterized by impairments in social reciprocal interaction, communication, and repetitive and stereotyped patterns of behavior, interests, and activities [1]. A number of neuropathologic abnormalities have been characterized in brains from individuals with autism including macrocephaly, volumetric and cellular abnormalities of the frontal cortex, parietal cortex, the limbic structures, and cerebellum, and cortical minicolumnar disorganization [2-4]. Recently, the Centers for Disease Control and Prevention (CDC) reported a prevalence for autism of 11.3 per 1,000 (one in 88) children aged eight years in the United States in 2008 [5]. This represents a 78% increase in incidence over the previously reported finding of 6.4 per 1,000 in 2002 [5].

Beyond core symptoms, people with autism display a number of comorbidities including seizure disorder, intellectual disability, and other cognitive impairments [6]. The presence of seizure disorder has been estimated from 5% to 40% [6]. Epileptiform activity has been shown to cause brief episodes of impaired cognitive function known as transitory cognitive impairment (TCI) [7,8] and may contribute to cognitive deficits in individuals with autism. Due to the heterogeneous nature of autism spectrum disorders, it is not surprising that multiple gene families have been implicated in the pathology of autism [9-11] with 50 gene or gene variants accounting for approximately 30% of autism spectrum cases [11]. Many of these candidate genes overlap with those of other disorders with autistic behavioral deficits including tuberous sclerosis [12], fragile X syndrome (FXS) [13], Rett syndrome [14], and Angelman syndrome [15].

There is a wide degree of overlap between behavioral deficits of autism and FXS, the most common inherited form of intellectual disability. Indeed, approximately 25% to 47% of people with FXS display a comorbid diagnosis of autism [16,17]. Similar to those diagnosed with autism, people diagnosed with FXS display learning deficits, delayed language acquisition, impaired motor skills, and repetitive behavior [18]. FXS is caused by mutations in the fragile X mental retardation 1 gene (*FMR1*) leading ultimately to the loss of fragile X mental retardation protein (FMRP) expression. FMRP binds approximately 4% of all mRNAs expressed in brain [19,20] and acts primarily as a translational repressor. FMRP is expressed in both glia and neurons [21,22] and in neurons is highly localized to the dendrites and spines [23-25].

FMRP acts as a negative regulator of group I metabotropic glutamate signaling, particularly metabotropic glutamate receptor 5 (mGluR5) and it is believed that runaway glutamatergic signaling, particularly in the dendrites, is ultimately responsible for the deficits associated with FXS [26]. An anatomical abnormality of neurons from brains of individuals with FXS, autism, and *Fmr1* knockout (KO) mice are dendrites with an overabundance of immature long, thin spines [27-29]. The profusion of dendritic spines could lead to an abnormally large amount of synapse formation and result in the cognitive impairments associated with FXS as well autism. In individuals with autism, greater spine density has been correlated with lower cognitive function [29].

We have previously observed reductions in FMRP in the cerebellar vermis and superior frontal cortex (Brodmann Area 9 (BA9)) of adults with autism and increased expression of mGluR5 in the vermis and BA9 of children with autism [30,31]. These represent the first findings of altered FMRP and mGluR5 in individuals with autism who do not have a comorbid diagnosis of FXS. In the current study, we have expanded upon our initial studies to examine protein expression of four known targets of FMRP and mGluR5 signaling that may play a role in regulating spine density, protein synthesis, and synaptic transmission: homer 1, amyloid beta A4 precursor protein (APP), ras-related C3 botulinum toxin substrate 1 (RAC1), and striatal-enriched protein tyrosine phosphatase (STEP) in the same brain regions. We hypothesized that targets of FMRP would display altered expression, further implicating this signaling pathway in the etiology of autism. Alterations in levels of these targets of FMRP could potentially be corrected therapeutically to ameliorate symptoms of autism.

## Methods

### Tissue preparation

The Institutional Review Board of the University of Minnesota, School of Medicine approved all experimental procedures for this study. Frozen postmortem blocks of the superior frontal cortex (BA9) and cerebellar vermis were obtained from the NICHD Brain and Tissue Bank for Developmental Disorders, University of Maryland, Baltimore, MD; the Harvard Brain Tissue Resource Center; the Brain Endowment Bank, Miami, Florida; and the Autism Tissue Program. The tissue samples (Table 1) were prepared as described previously [30,31] and each sample included both grey and white matter. None of the controls had a history of neuropsychiatric disorders, seizure disorder, or intellectual disability.

**Table 1:** Demographic data for individuals with autism and controls

**Case**	**Dx**	**Sex**	**Age**	**PMI (hrs)**	**Ethnicity**	**Medication history**	**Cause of death**	**Seizure**	**ID**	**Side of brain**	**Brain region**
UMB-4670	Control	M	4	17	Caucasian	None	*Commotio cordis*	No	No	Unknown	BA9, Vermis
UMB-4898	Control	M	7	12	Caucasian	Methylphenidate; clonidine	Drowning	No	No	Unknown	Vermis
UMB-1674	Control	M	8	36	Caucasian	None	Drowning	No	No	Unknown	BA9, Vermis
UMB-4787	Control	M	12	15	African American	Montelukast; albuteral; prednisone, loratadine	Asthma	No	No	Unknown	BA9, Vermis
UMB-1823	Control	M	15	18	Caucasian	None	MVA	No	No	Unknown	BA9, Vermis
AN17425	Control	M	16	26.16	Unknown	None	Heart attack	No	No	Left	BA9
AN15105	Control	M	18	19.83	Unknown	None	Unknown	No	No	Unknown	BA9, Vermis
AN03217	Control	M	19	18.58	Caucasian	None	Pneumonia	No	No	Left	BA9
UMB-1846	Control	F	20	9	Caucasian	None	MVA	No	No	Unknown	BA9, Vermis
AN07176	Control	M	21	29.91	Unknown	None	MVA	No	No	Left	BA9
AN14368	Control	M	22	24.2	Unknown	None	Unknown	No	No	Left	BA9
AN19760	Control	M	28	23.25	Unknown	None	Unknown	No	No	Left	BA9
AN15566	Control	F	32	28.92	Unknown	None	Unknown	No	No	Left	BA9, Vermis
UMB-1169	Control	M	33	27	African American	Metoclopramide; loratadine	Dilated cardiomyopathy (morbid obesity)	No	No	Unknown	BA9, Vermis
UMB-1376	Control	M	37	12	African American	None	ASCVD	No	No	Unknown	BA9, Vermis
AN15151	Control	M	41	30.4	Unknown	None	Heart attack	No	No	Unknown	BA9, Vermis
AN19440	Control	F	50	20.25	Unknown	None	Heart attack	No	No	Unknown	BA9
AN12240	Control	M	51	4.75	Caucasian	None	Heart attack	No	No	Right	BA9
AN13295	Control	M	56	22.12	Unknown	None	Unknown	No	No	Right	BA9
AN08873	Autism	M	5	25.5	Caucasian	None	Drowning	No	No	Left	BA9
AN13872	Autism	F	5	32.73	Asian	None	Drowning	No	No	Left	BA9, Vermis
UMB-1349	Autism	M	5	39	Caucasian	None	Drowning	No	No	Unknown	Vermis
UMB-1174	Autism	F	7	14	Caucasian	None	Seizure disorder	Yes	No	Unknown	BA9
UMB-4231	Autism	M	8	12	African American	Olanzapine; galantamine	Drowning	No	Yes	Unknown	BA9, Vermis
AN19511	Autism	M	8	22.16	Caucasian	None	Cancer	Yes	No	Left	BA9, Vermis
UMB-4721	Autism	M	8	16	African American	None	Drowning	No	No	Unknown	Vermis
AN16641	Autism	M	9	27	Caucasian	Carbamazepine; methylphenidate; clonidine	Seizure disorder	Yes	Yes	Left	BA9
AN16115	Autism	F	11	12.88	Caucasian	Carbamazepine; lamotrigine; dextroamphetamine; topiramate	Seizure/drowning	Yes	Yes	Right	Vermis
UMB-4899	Autism	M	14	9	Caucasian	None	Drowning	No	No	Unknown	BA9, Vermis
AN17138	Autism	M	16	24	Asian	Fexofenadine; buspirone; topiramate	Seizure disorder	Yes	No	Left	BA9, Vermis
AN01570	Autism	F	18	6.75	Caucasian	None	Seizure disorder	Yes	No	Right	BA9
AN00764	Autism	M	20	23.66	Caucasian	Erythromycin gel; minocycline	MVA	No	Yes	Left	BA9
UMB-1638	Autism	F	20	50	Caucasian	None	Seizure disorder	Yes	Yes	Unknown	BA9
AN09730	Autism	M	22	25	Caucasian	Aripiprazole; lamotrigine; zonisamide	Aspiration	Yes	No	Left	BA9, Vermis
AN08166	Autism	M	29	43.25	Caucasian	Fexofenadine; ziprasidone HCl; carbamazepine	Seizure (suspected)	Yes	No	Left	BA9, Vermis
AN12457	Autism	F	29	17.83	Caucasian	Fluvoxamine	Seizure disorder	Yes	No	Left	BA9, Vermis
AN11989	Autism	M	30	16.06	Caucasian	None	Heart failure (congestive)	No	No	Left	BA9
AN08792	Autism	M	30	20.33	Caucasian	Cisapride; clorazepate; sodium valproate; phenytoin; folic acid; primidone; phenobarbital; omeprazole; metoclopramide	Gastrointestinal bleeding	Yes	No	Left	BA9, Vermis
UMB-5027	Autism	M	37	26	African American	None	Obstruction of bowel due to adhesion	No	No	Unknown	BA9, Vermis
AN06420	Autism	M	39	13.95	Caucasian	None	Cardiac tamponade	No	No	Left	BA9, Vermis
AN14613	Autism	M	39	22.75	Caucasian	None	Sudden unexpected death	Yes	No	Unknown	BA9
AN17777	Autism	F	49	16.33	Caucasian	Wafarin; venlafaxine; erythromycin; lansoprazole; risperidone; metformin; gabapentin; propranolol; levothyroxine	Pulmonary arrest	No	Yes	Left	BA9, Vermis
AN01093	Autism	M	56	19.48	Caucasian	Benztropine mesylate; haloperidol; lithium; chlorpromazine; alprazolam	Anoxic encephalopathy	Yes	No	Right	BA9, Vermis
**Children**		**Control**			**Autistic**		**Change**	***P*-value**		***d***	
Age ± SD (years)		11.4 ± 5.22			9.5 ± 4.38		↓16.6%	ns		nd	
PMI ± SD (years)		20.6 ± 8.1			20.1 ± 9.94		↓2.4%	ns		nd	
Gender		7M			8M:4F		nd	nd		nd	
**Adults**		**Control**			**Autistic**		**Change**	***P-*value**		***d***	
Age ± SD (years)		34.2 ± 13			33.3 ± 11.2		↓2.6%	ns		nd	
PMI ± SD (years)		20.9 ± 8.41			24.6 ± 11.1		↑17.7%	ns		nd	
Gender		9M:3F			9M:3F		nd	nd		nd	

*ASCVD*, Arteriosclerotic cardiovascular disease; *Dx*, Diagnosis; *Hrs*, Hours; *PMI*, Postmortem interval; *M*, Male; *F*, Female; *EtOH*, Alcohol; *ID*, Intellectual disability; *MVA*, Motor vehicle accident; *nd*, Not determined; *ns*, Not significant; *d*, Cohen’s test statistic.

### Sodium dodecyl sulfate polyacrylamide gel electrophoresis (SDS-PAGE) and western blot analysis

Tissue samples from the cerebellar vermis (vermal lobule unknown) (in adults, n = 5 controls and 8 autistic adults; in children, n = 6 controls and 8 autistic children) and BA9 (in adults, n = 12 controls and 12 autistic adults; in children, n = 6 controls and 9 autistic children) were prepared. Children were defined as those younger than 13 years of age; adolescents were defined as those between the ages of 13 years to 18 years; and adults were defined as those 19 years of age or older. For this study, children and adolescents were grouped together. Samples were mixed with denaturing SDS sample buffer and denatured by heating at 100°C for 5 minutes. SDS-PAGE gels were prepared using standard Laemmli solutions. For experiments involving RAC1 or STEP, 12% resolving gels were used; 10% resolving gels were used for experiments involving homer 1, neuronal specific enolase (NSE), or β-actin, and for experiments involving APP, 6% resolving gels were used. In all cases, 5% stacking gels were used. Thirty μg of protein per lane was loaded onto the gel and electrophoresed for 15 minutes at 75 V followed by 55 minutes at 150 V at room temperature (RT). We minimized interblot variability by including samples from subjects of each group (control and autism) on each gel. Samples were run in duplicate. Proteins were electroblotted onto nitrocellulose membranes for 2 hrs at 300 mAmp at 4°C as previously described [30,31]. Blots were blocked with 0.2% I-Block (Tropix, Bedford, MA, USA) in PBS with 0.3% Tween 20 for one hour at RT followed by an overnight incubation in primary antibodies at 4°C. The primary antibodies used were: anti-RAC1 (1:500; BD Transduction, San Jose, CA, USA), anti-APP (1:250; Abcam Inc., Cambridge, MA, USA), anti-homer 1 (1:500; Abnova, Taipei, Taiwan), anti-STEP (1:200; Abgent, San Diego, CA, USA), anti-NSE (1:2,000; Abcam Inc.), and anti-β-actin (1:5,000; Sigma Aldrich, St. Louis, MO, USA). Blots were washed for 30 minutes and incubated in secondary antibodies for one hour at RT as previously described [30,31]. Secondary antibodies were goat anti-rabbit IgG (A9169, Sigma Aldrich, 1:80,000) or goat anti-mouse IgG (A9044, Sigma Aldrich, 1:80,000). Blots were washed twice in PBS supplemented with Tween-20 (PBST) and bands were visualized using the ECL plus detection system (GE Healthcare, Little Chalfont, Buckinghamshire, UK) and exposed to CL-Xposure film (Thermo Scientific, Rockford, IL, USA). The molecular weights of approximately 120 kDa and 88 kDa (APP); 66 kDa, 61 kDa, 46 kDa, 33 kDa, and 27 kDa (STEP); 46 kDa (NSE); 45 kDa (homer 1); 42 kDa (β-actin); and 21 kDa (RAC1) immunoreactive bands were quantified with background subtraction using a RioRad densitometer and BioRad MultiAnalyst software (Bio-Rad, Hercules, CA, USA). Results obtained were based on two to four independent experiments.

### Statistical analysis

Statistical analysis of protein data was performed as previously described [30,32-34]. The independent samples *t*-test was used to compare people with autism to healthy controls. Separate analyses were conducted for children and adults. Analysis of covariance was used to estimate the effect of covariates on group comparisons. Effect sizes were calculated using Cohen’s *d* statistic, with values >0.8 considered a large effect [35]. For analysis of confounders, significance was set at *P* <0.06.

## Results

**Figure 1:**
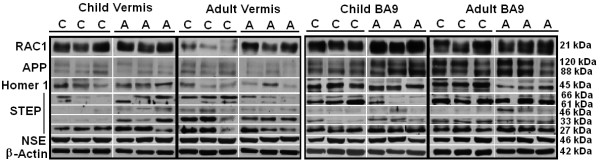
**Representative samples of RAC1, APP, Homer 1, STEP, NSE, and β-actin from the cerebellar vermis and BA9 from controls and people with autism.** C, controls; A, people with autism; RAC1, Ras-related C3 botulinum toxin substrate 1; APP, amyloid beta A4 precursor protein; STEP, striatal-enriched protein tyrosine phosphatase; NSE, neuronal specific enolase; BA9, Brodmann Area 9.

**Figure 2:**
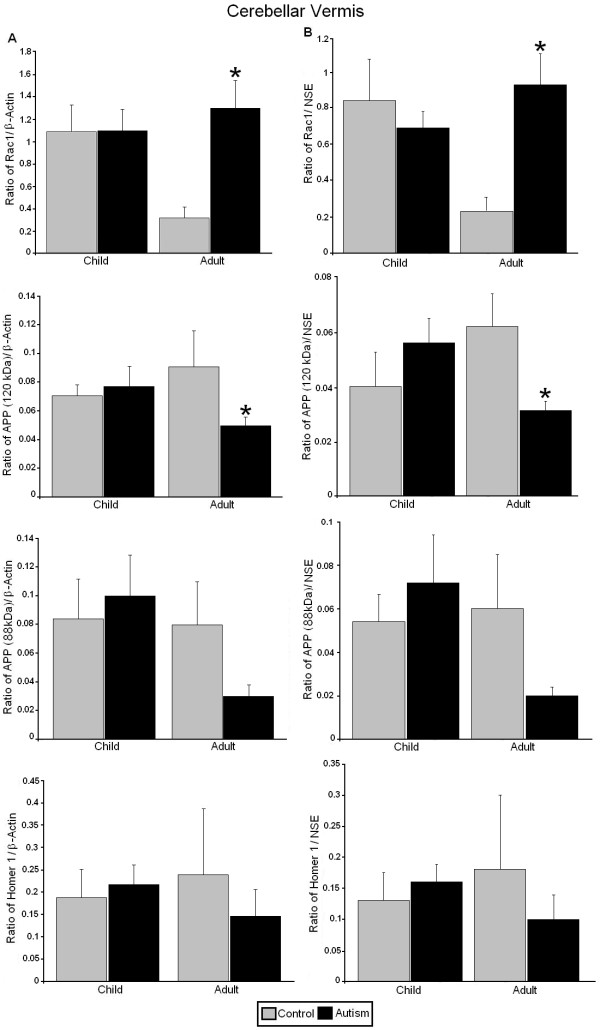
**Expression of RAC1, APP, and homer 1 in the cerebellar vermis of subjects with autism and controls. (A)** Mean RAC1/β-actin, APP 120 kDa/β-actin, APP 88/β-actin, and homer 1/β-actin ratios for controls (gray histogram bars) and people with autism (black histogram bars) are shown for the cerebellar vermis. **(B)** Mean RAC1/NSE, APP 120 kDa/NSE, APP 88/NSE, and homer 1/NSE ratios for controls (gray histogram bars) and people with autism (black histogram bars) are shown for the cerebellar vermis. Error bars express standard error of the mean. ^*^P <0.05. RAC1, Ras-related C3 botulinum toxin substrate 1; amyloid beta A4 precursor protein; NSE, neuronal specific enolase.

**Figure 3:**
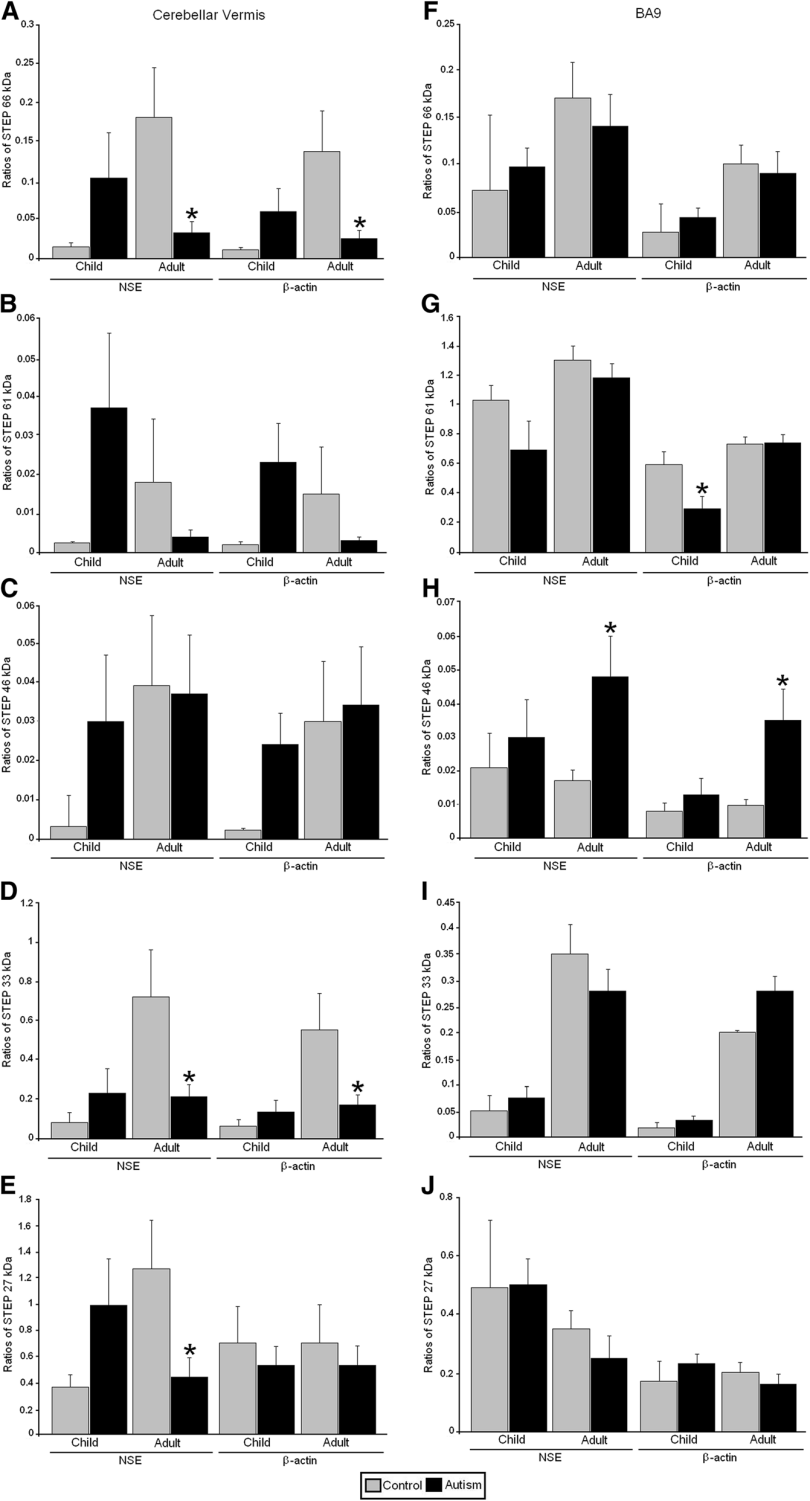
**Expression of STEP in the cerebellar vermis and BA9 of subjects with autism and controls.** Control group data are presented as gray histogram bars; autistic group data are presented as black histogram bars. **(A)** and **(F)** STEP 66 kDa. **(B)** and **(G)** STEP 61 kDa. **(C)** and **(H)** STEP 46 kDa. **(D)** and **(I)**. STEP 33 kDa. **(F)** and **(J)** STEP 27 kDa. Error bars express standard error of the mean. ^*^P <0.05. STEP, striatal-enriched protein tyrosine phosphatase; NSE, neuronal specific enolase; BA9, Brodmann Area 9.

All western blotting results were normalized against β-actin and NSE and are expressed as ratios to β-actin and NSE. In the cerebellar vermis of adults with autism we observed significantly increased expression of the RAC1/β-actin ratio (*P* <0.025, *d* = 1.62) and the RAC1/NSE ratio (*P* <0.016, *d* = 1.73) (Figures 1 and 2, Table 2). We also observed a significant reduction in expression of the APP 120 kDa/β-actin ratio (*P* <0.018, *d* = −2.04) and the APP 120 kDa/NSE ratio (*P* <0.012, *d* = −2.40) (Figures 1 and 2, Table 2). Ratios for STEP 66 kDa/β-actin (*P* <0.023, *d* = −1.51), STEP 66 kDa/NSE (*P* <0.018, *d* = −1.59), STEP 33 kDa/β-actin (*P* <0.024, *d* = −1.63) and STEP 33 kDa/NSE (*P* <0.020, *d* = −1.68) were significantly reduced in the cerebellar vermis of adults with autism (Figures 1 and 3, Table 2). STEP 27 kDa/NSE was also significantly reduced in the cerebellar vermis of subjects with autism (*P* <0.038, *d* = −1.40) (Table 2). We did not observe alterations in levels of homer 1 or APP 88 kDa in the cerebellar vermis of adults with autism and there were no significant changes for any of the proteins in the cerebellar vermis of children with autism (Figures 1, 2, 3, Table 2).

**Table 2:** **Western blotting results for RAC1, homer 1, APP, STEP, NSE, and β-actin and their ratios in the cerebellar vermis**^**a**^

	**Control**	**Autistic**	**Change**	***P*-value**	**Cohen’s *d***
**Adults**					
RAC1/β-actin	0.315 ± 0.206	1.3 ± 0.714	313%↑	0.025^b^	1.62^b^
Homer 1/β-actin	0.237 ± 0.303	0.147 ± 0.147	38%↓	ns	0.42
APP 120 kDa/β-actin	0.091 ± 0.029	0.050 ± 0.016	45%↓	0.018^b^	−2.04^b^
APP 88 kDa/β-actin	0.08 ± 0.06	0.03 ± 0.02	63%↓	ns	1.26
STEP 66 kDa/β-actin	0.136 ± 0.116	0.025 ± 0.029	82%↓	0.023^b^	−1.51^b^
STEP 61 kDa/β-actin	0.015 ± 0.028	0.003 ± 0.004	80%↓	ns	−0.67
STEP 46 kDa/β-actin	0.03 ± 0.034	0.034 ± 0.041	13%↑	ns	0.11
STEP 33 kDa/β-actin	0.55 ± 0.38	0.17 ± 0.13	69%↓	0.024^b^	−1.63^b^
STEP 27 kDa/β-actin	0.70 ± 0.67	0.53 ± 0.39	24%↓	ns	−0.32
β-actin	10.2 ± 1.67	9.06 ± 3.1	11%↓	ns	nd
**Children**					
RAC1/β-actin	1.09 ± 0.545	1.1 ± 0.471	0.9%↑	ns	0.01
Homer 1/β-actin	0.188 ± 0.13	0.218 ± 0.109	16%↑	ns	0.26
APP 120 kDa/β-actin	0.071 ± 0.029	0.077 ± 0.033	8.4%↑	ns	0.19
APP 88 kDa/β-actin	0.084 ± 0.055	0.100 ± 0.071	19%↑	ns	0.25
STEP 66 kDa/β-actin	0.01 ± 0.008	0.059 ± 0.091	490%↑	ns	0.64
STEP 61 kDa/β-actin	0.002 ± 0.002	0.023 ± 0.031	1050%↑	ns	1.15
STEP 46 kDa/β-actin	0.0022 ± 0.0011	0.024 ± 0.024	991%↑	ns	1.16
STEP 33 kDa/β-actin	0.057 ± 0.085	0.13 ± 0.17	128%↑	ns	0.51
STEP 27 kDa/β-actin	0.70 ± 0.68	0.53 ± 0.38	24%↓	ns	−0.72
β-actin	9.48 ± 1.11	9.52 ± 3.71	0.4%↑	ns	nd
**Adults**					
RAC1/NSE	0.23 ± 0.16	0.93 ± 0.49	304%↑	0.016^b^	1.73^b^
Homer 1/NSE	0.18 ± 0.24	0.099 ± 0.11	45%↓	ns	−0.46
APP 120 kDa/NSE	0.062 ± 0.021	0.031 ± 0.008	50%↓	0.012^b^	−2.40^b^
APP 88 kDa/NSE	0.06 ± 0.05	0.02 ± 0.01	67%↓	ns	−1.22
STEP 66 kDa/NSE	0.18 ± 0.14	0.032 ± 0.04	82%↓	0.018^b^	−1.59^b^
STEP 61 kDa/NSE	0.018 ± 0.035	0.004 ± 0.005	78%↓	ns	−0.21
STEP 46 kDa/NSE	0.039 ± 0.041	0.037 ± 0.042	5.1%↓	ns	−0.59
STEP 33 kDa/NSE	0.72 ± 0.49	0.21 ± 0.17	71%↓	0.020^b^	−1.68^b^
STEP 27 kDa/NSE	1.27 ± 0.82	0.44 ± 0.38	65%↓	0.038^b^	−1.40^b^
NSE	7.42 ± 1.26	7.69 ± 1.3	3.6%↑	ns	nd
**Children**					
RAC1/NSE	0.84 ± 0.51	0.69 ± 0.23	18%↓	ns	−0.38
Homer 1/NSE	0.13 ± 0.09	0.16 ± 0.07	23%↑	ns	0.33
APP 120 kDa/NSE	0.040 ± 0.025	0.056 ± 0.021	40%↑	ns	0.70
APP 88 kDa/NSE	0.054 ± 0.025	0.072 ± 0.053	33%↑	ns	0.42
STEP 66 kDa/NSE	0.014 ± 0.011	0.102 ± 0.165	628%↑	ns	0.61
STEP 61 kDa/NSE	0.0024 ± 0.001	0.037 ± 0.054	1441%↑	ns	0.96
STEP 46 kDa/NSE	0.003 ± 0.002	0.03 ± 0.047	900%↑	ns	1.02
STEP 33 kDa/NSE	0.078 ± 0.12	0.23 ± 0.33	194%↑	ns	0.55
STEP 27 kDa/NSE	0.36 ± 0.24	0.99 ± 0.93	175%↑	ns	0.77
NSE	6.97 ± 0.84	6.23 ± 1.53	10.6%↓	ns	nd

Values for control and autistic groups are presented as mean ± SD ^a^RAC1, ras-related C3 botulinum toxin substrate 1; APP, amyloid beta A4 precursor protein; STEP, striatal-enriched protein tyrosine phosphatase; NSE, neuronal specific enolase; nd, not determined; ns, not significant. ^b^Statistically significant.

**Figure 4:**
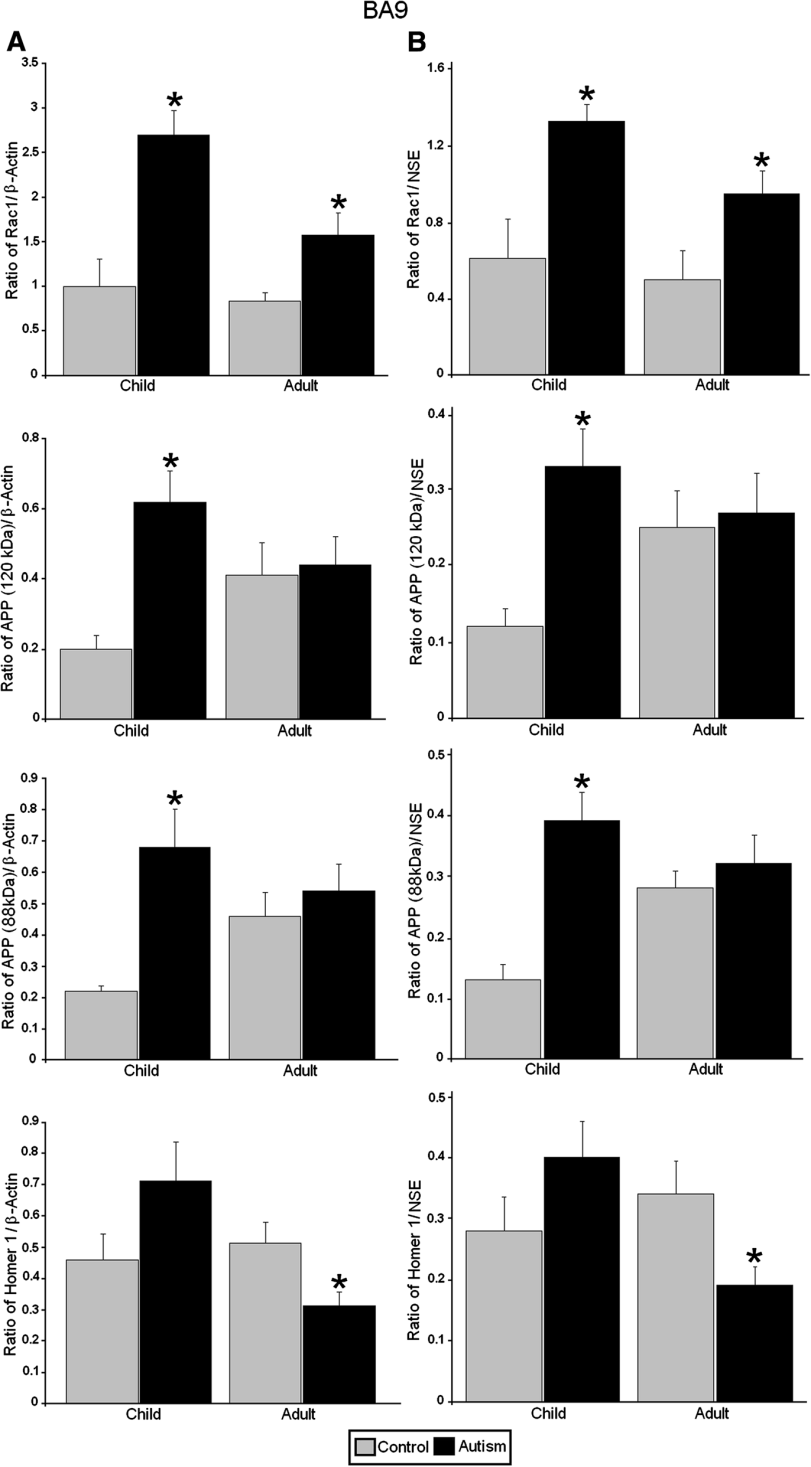
**Expression of RAC1, APP, and homer 1 in BA9 of subjects with autism and controls. (A)** Mean RAC1/β-actin, APP 120 kDa/β-actin, APP 88/β-actin, and homer 1/β-actin ratios for controls (gray histogram bars) and people with autism (black histogram bars) are shown for BA9. **(B)** Mean RAC1/NSE, APP 120 kDa/NSE, APP 88/NSE, and homer 1/NSE ratios for controls (gray histogram bars) and people with autism (black histogram bars) are shown for BA9. Error bars express standard error of the mean. ^*^P <0.05. RAC1, Ras-related C3 botulinum toxin substrate 1; amyloid beta A4 precursor protein; NSE, neuronal specific enolase; BA9, Brodmann Area 9.

In BA9 of adults with autism we observed significantly increased ratios for RAC1/β-actin (*P* <0.031, *d* = 1.32), RAC1/NSE (*P* <0.042, *d* = 1.24) and significantly reduced ratios for homer 1/β-actin (*P* <0.027, *d* = −1.37), homer 1/NSE (*P* <0.020, *d* = −1.43) (Figures 1 and 4, Table 3). STEP 46 kDa/β-actin ratio (*P* <0.012, *d* = 1.10) and STEP 46 kDa/NSE ratio (*P* <0.020, *d* = 1.02) were significantly increased in BA9 of adults with autism (Figures 1 and 3, Table 3). In BA9 of children with autism we observed significant increases in ratios for RAC1/β-actin (*P* <0.008, *d* = 2.74), RAC1/NSE (*P* <0.017, *d* = 2.09), APP 120 kDa/β-actin (*P* <0.032, *d* = 2.03), APP 120 kDa/NSE (*P* <0.017, *d* = 2.12), APP 88 kDa/β-actin (*P* <0.025, *d* = 2.54) and APP 88 kDa/NSE (*P* <0.012, *d* = 3.24) (Figures 1 and 4, Table 3). Finally, there was a significant reduction in STEP 61 kDa/β-actin ratio (*P* <0.036, *d* = −1.23) in BA9 of children with autism (Figures 1 and 3, Table 3).

**Table 3:** **Western blotting results for RAC1, homer 1, APP, STEP, NSE, and β-actin and their ratios in BA9**^**a**^

	**Control**	**Autistic**	**Change**	***P*-value**	**Cohen’s *d***
**Adults**					
RAC1/β-actin	0.834 ± 0.554	1.66 ± 0.65	99%↑	0.031^b^	1.32^b^
Homer 1/β-actin	0.512 ± 0.152	0.324± 0.132	37%↓	0.027^b^	−1.37^b^
APP 120 kDa/β-actin	0.41 ± 0.23	0.45 ± 0.24	9.8%↑	ns	0.17
APP 88 kDa/β-actin	0.46 ± 0.19	0.53 ± 0.24	15%↑	ns	0.29
STEP 66 kDa/β-actin	0.10 ± 0.07	0.09 ± 0.08	10%↓	ns	−0.11
STEP 61 kDa/β-actin	0.73 ± 0.17	0.74 ± 0.18	1.3%↑	ns	0.08
STEP 46 kDa/β-actin	0.0097 ± 0.006	0.035 ± 0.031	261%↑	0.012^b^	1.10
STEP 33 kDa/β-actin	0.20 ± 0.13	0.18 ± 0.09	10%↓	ns	−0.25
STEP 27 kDa/β-actin	0.20 ± 0.12	0.16 ± 0.12	20%↓	ns	−0.29
β-actin	13.2 ± 2.41	13.1 ± 2.31	0.75%↓	ns	nd
**Children**					
RAC1/β-actin	1 ± 0.616	2.8 ± 0.7	180%↑	0.008^b^	2.74^b^
Homer 1/β-actin	0.46 ± 0.17	0.77 ± 0.31	67%↑	ns	1.21
APP 120 kDa/β-actin	0.20 ± 0.07	0.63 ± 0.25	215%↑	0.032^b^	2.03^b^
APP 88 kDa/β-actin	0.22 ± 0.03	0.75 ± 0.26	241%↑	0.025^b^	2.54^b^
STEP 66 kDa/β-actin	0.027 ± 0.026	0.043 ± 0.031	59%↑	ns	0.54
STEP 61 kDa/β-actin	0.59 ± 0.22	0.29 ± 0.24	51%↓	0.036^b^	−1.23^b^
STEP 46 kDa/β-actin	0.008 ± 0.006	0.013 ± 0.014	62%↑	ns	0.45
STEP 33 kDa/β-actin	0.017 ± 0.026	0.033 ± 0.023	94%↑	ns	0.64
STEP 27 kDa/β-actin	0.17 ± 0.17	0.23 ± 0.10	35%↑	ns	0.45
β-actin	11.5 ± 1.31	11.7 ± 2.05	1.7%↑	ns	nd
**Adults**					
RAC1/NSE	0.50 ± 0.33	0.95 ± 0.37	90%↑	0.042^b^	1.24
Homer 1/NSE	0.34 ± 0.12	0.19 ± 0.10	44%↓	0.020^b^	−1.43
APP 120 kDa/NSE	0.25 ± 0.12	0.27 ± 0.16	8%↑	ns	0.16
APP 88 kDa/NSE	0.28 ± 0.07	0.32 ± 0.14	14%↑	ns	0.27
STEP 66 kDa/NSE	0.17 ± 0.13	0.14 ± 0.12	18%↓	ns	−0.35
STEP 61 kDa/NSE	1.3 ± 0.34	1.18 ± 0.33	9.2%↓	ns	−0.25
STEP 46 kDa/NSE	0.017 ± 0.011	0.048 ± 0.041	182%↑	0.020^b^	1.02^b^
STEP 33 kDa/NSE	0.35 ± 0.20	0.28 ± 0.14	20%↓	ns	−0.46
STEP 27 kDa/NSE	0.35 ± 0.21	0.25 ± 0.17	29%↓	ns	−0.53
NSE	9.14 ± 3.73	8.59 ± 1.41	6%↓	ns	nd
**Children**					
RAC1/NSE	0.61 ± 0.42	1.33 ± 0.21	118%↑	0.017^b^	2.09^b^
Homer 1/NSE	0.28 ± 0.11	0.40 ± 0.15	43%↑	ns	0.94
APP 120 kDa/NSE	0.12 ± 0.04	0.33 ± 0.12	175%↑	0.017^b^	2.12^b^
APP 88 kDa/NSE	0.13 ± 0.004	0.39 ± 0.11	200%↑	0.012^b^	3.24^b^
STEP 66 kDa/NSE	0.072 ± 0.081	0.097 ± 0.072	34%↑	ns	0.33
STEP 61 kDa/NSE	1.03 ± 0.25	0.69 ± 0.60	33%↓	ns	−0.68
STEP 46 kDa/NSE	0.021 ± 0.021	0.030 ± 0.033	43%↑	ns	0.37
STEP 33 kDa/NSE	0.050 ± 0.083	0.075 ± 0.054	50%↑	ns	0.37
STEP 27 kDa/NSE	0.49 ± 0.57	0.50 ± 0.26	2%↑	ns	0.031
NSE	6.58 ± 2.48	5.63 ± 1.3	14%↓	ns	nd

Values for control and autistic groups are presented as mean ± SD.^a^RAC1, ras-related C3 botulinum toxin substrate 1; APP, amyloid beta A4 precursor protein; STEP, striatal-enriched protein tyrosine phosphatase; NSE, neuronal specific enolase; BA9, Brodmann’s area 9; nd, not determined; ns, not significant. ^b^Statistically significant.

In adults, we examined ethnicity, postmortem interval (PMI), intellectual disability, history of seizures, antidepressant, antipsychotic drug (APD), and anticonvulsant use in relation to the outcome measures. PMI, gender and intellectual disability were not significantly related to any of the values expressed as ratios of β-actin or NSE. Adults using anticonvulsants displayed significantly higher STEP 46 kDa/β-actin than all adults who did not use anticonvulsants (including autistic as well as nonautistic) (t(22) = 5.23, *P* <0.001). Adults with autism who were taking anticonvulsants displayed significantly increased expression of STEP 46 kDa/β-actin versus adults with autism who were not taking anticonvulsants (t(10) = 2.85, *P* <0.017). Moreover, adults with autism who were taking anticonvulsants displayed significantly increased expression of STEP 46 kDa/β-actin than adult controls (t(15) = 5.28, *P* <0.001). Finally, there was no significant difference in STEP 46 kDa/β-actin when adult controls were compared against adults with autism who were not taking anticonvulsants (t(17) = 1.44, *P* <0.17). Increased STEP 46 kDa expression was only seen in ratios compared to β-actin but not NSE, indicating that the difference was not specific to neuronal cells. By the same token, STEP 46 kDa/β-actin value in BA9 was also significantly different in those with seizure history versus those without (t(22) = 3.00, *P* <0.007). However, no significant difference existed between values in adults with autism with seizure disorder versus adults with autism who did not have seizure disorder, nullifying this association (mean 0.043 ± 0.034 versus 0.023 ± 0.027, *P* <0.29). Adults with autism who were taking anticonvulsants displayed significantly lower homer 1/NSE in BA9 than controls and adults with autism who were not taking anticonvulsants (t(15) = 2.69, *P* <0.017). While a comparison of homer 1/NSE values between adults with autism who took anticonvulsants (mean of 0.13 ± 0.086) versus those who did not (mean of 0.23 ± 0.085) did not show a significant difference (*P* <0.063), it is possible that the reduction of homer 1/NSE is at least partly due to anticonvulsant use.

For those using APDs, there were significantly reduced expression of APP 120 kDa/NSE in BA9 (t(15) = 2.20, *P* <0.044), APP 88 kDa/NSE in BA9 (t(14) = 2.63, *P* <0.02), and STEP 33 kDa/β-actin (t(22) = 2.18, *P* <0.040). However, none of these values showed any significant changes in adults with autism when compared with controls (*P* <0.8, *P* <0.55, and *P* <0.55, respectively). STEP 46 kDa/β-actin was significantly higher (t(22) = 2.79, *P* <0.011) in BA9 in subjects who took APD medication than in those who did not. However, comparison of values for STEP 46 kDa/β-actin in BA9 between subjects with autism taking APDs versus subjects with autism who did not take APDs was not statistically significant (mean 0.051 ± 0.031 versus 0.027 ± 0.03, respectively, *P* <0.22). For antidepressant use, the single individual taking antidepressants displayed significantly higher expression of APP 88 kDa/NSE in BA9 than the others (t(14) = 2.51, *P* <0.025) but this difference only reflects change in one subject and is not extendable to the whole group and thus, not amenable to statistical testing. There was a confounding effect of gender on homer 1/NSE in BA9 of adults (t(14) = 2.04, *P* <0.060). There was a significant reduction in homer 1/NSE in BA9 of adults with autism versus controls (*P* <0.020) (see Table 3). The mean for male subjects with autism was 0.262 ± 0.081 and the mean for female subjects with autism was 0.417 ± 0.15 (*P* <0.061), which was not significantly different. Due to having only two subjects for comparison, we were unable to compare female controls with female autistic subjects. Comparing male controls to male autistic subjects resulted in a significant reduction, similar to that reported in Table 3. It is therefore unlikely that gender has a meaningful effect on homer 1/NSE values. There was also a significantly higher expression of RAC1/NSE in Caucasian than African American subjects in BA9 (t(11) = 2.86, *P* <0.016). However, this comparison is not relevant as the African American sample size numbered only two subjects. Finally, there was an impact of anticonvulsant use on APP 88 kDa/NSE in BA9 of adults (t(14) = 2.12, *P* <0.052). However, there was no significant difference between adults with autism versus controls for APP 88 kDa/NSE in BA9 of adults, so this is not meaningful.

For children, ethnicity (Caucasian versus African American), history of seizures, antidepressant, APD, and anticonvulsant use were not significantly related to any of the β-actin or NSE measures. There was significant positive correlation between PMI and APP 88 kDa/NSE in BA9 of children (*R* = 0.734, *P* <0.060). However, PMI was not significantly different between control or autistic children in BA9, so this finding is not meaningful. For gender, one female subject displayed significantly higher APP 120 kDa/NSE in BA9 than male subjects (t(6) = 3.16, *P* <0.02). However, this significance could not be linked to a group of subjects, and thus, was not amenable to further statistical testing. Those with intellectual disability had significantly higher ratios on STEP 46 kDa/β-actin BA9 (t(13) = 2.81, *P* <0.015]. However, STEP 46 kDa/β-actin in BA9 was not significantly different between controls and those with autism.

To further investigate the effect of medications on our results we performed a second analysis comparing controls to people with autism who were not taking medication (Additional files 1: Table S1 and Additional file 2: Table S2). In the cerebellar vermis of adults, RAC1/NSE and RAC1/β-actin remained significant (Additional file 1: Table S1). In BA9 of adults, RAC1/NSE and RAC1/β-actin remained significant as did RAC1/β-actin in children along with APP 120 kDa/NSE, APP 120 kDa/β-actin, APP 88 kDa/NSE and APP 88 kDa/β-actin (Additional file 2: Table S2). Omitting people with autism who were on medication reduced the total number available for analysis so that seven comparisons could no longer be made, and in fifteen comparisons with a sample of three subjects for people with autism, power was reduced thus, affecting statistical significance.

Finally, we tested to see if there were statistical correlations between levels of FMRP or mGluR5 that we had previously reported [30,31] and levels of RAC1, STEP 66 kDa, STEP 61 kDa, STEP 46 kDa, STEP 33 kDa, STEP 27 kDa, APP120 kDa, APP 88 kDa, or homer 1. We did not find any significant statistical correlation for either FMRP or mGluR5 with any of the targets investigated in the current study (data not shown).

## Discussion

The current studies demonstrated abnormal expression of several downstream biochemical targets of FMRP and mGluR5-mediated signaling in the brains of children and adults with autism. The most salient results included: 1) upregulation of RAC1 in BA9 and the vermis of adults with autism, and in BA9 only among children with autism; 2) upregulation of APP120 and 88 kDa species in BA9 of children with autism and downregulation of APP 120 kDa in the vermis of adults with autism; 3) upregulation of STEP 46 kDa in BA9 and downregulation of STEP 66 kDa, 33 kDa, and 27 kDa in the vermis of adults with autism; 4) downregulation of STEP 61 kDa in BA9 of children with autism; and 5) downregulation of homer 1 in BA9 of adults with autism. We observed more significant effects in BA9 than in the cerebellar vermis, consistent with previous findings in autism [36]. The results summarized above will be discussed further and the significant involvement of these proteins in autism will be analyzed in the following paragraphs.

RAC1 is a member of the Rho family of GTPases, a subfamily of the RAS superfamily of small guanine triphosphate (GTP)-binding proteins. Molecules in this superfamily regulate a number of cellular processes including kinase activation, cytoskeletal rearrangement, cell growth, differentiation, and survival [37]. Members of the Rho family have been implicated in cell proliferation, apoptosis, and regulation of gene expression [37]. RAC1 is involved in remodeling of the actin cytoskeleton [38] and plays an important role in the formation and maturation of dendritic spines as well as regulation of spine density and morphology [39-43]. Mutations in the Rho GTPase pathway and their regulators have been involved in intellectual disability-related disorders [43]. For example, RAC1 partners with oligophrenin-1 and synaptojanin which have been linked to X-linked intellectual disability and Down’s syndrome, respectively [43-45]. Overexpression of RAC1 in cultured hippocampal neurons younger than 11 days *in vitro* resulted in the formation of dendritic spines, clustering of 2-amino-3-(5-methyl-3-oxo-1,2- oxazol-4-yl)propanoic acid (AMPA) receptors and increased the amplitude of miniature excitatory postsynaptic currents, suggesting that RAC1 also enhances excitatory synaptic transmission [42].

RAC1 has been shown to interact with FMRP via cytoplasmic FMRP interacting proteins (CYFIP1/2) [46,47]. RAC1-induced actin remodeling is altered in mouse fibroblasts that lack FMRP, suggesting a role for FMRP in modulating actin dynamics [48]. More recently, RAC1 expression has been demonstrated to be increased in the hippocampus, cortex, brainstem, and cerebella of *Fmr1* KO mice suggesting that FMRP acts as a negative regulator of RAC1 [49]. Our results of increased expression of RAC1 in the cerebellar vermis and BA9 of adults with autism, and in BA9 of children with autism, are novel and have never previously been reported in human postmortem brains of subjects with autism; they may be the result of reduced expression of FMRP in the same regions [30,31], and they mirror the same findings in *Fmr1* KO mice. The overexpression of RAC1, particularly in BA9, may suggest increased excitatory signaling, which could impact synaptic transmission and ultimately cognitive function. This is especially true as studies using transgenic mice with a constitutively active form of RAC1 show overproduction of small abnormal supernumerary spines [43,50,51], indicating that overproduction of RAC1 in BA9 of both children and adults with autism is evidence of overabundance of active and abnormal neuronal spines in the brains of people with autism examined in this study. There is evidence in the literature that RAC1 may stimulate stellation of primary astrocytes [52] and increase migration of astrocytes [53]. Additionally, some residual RAC1 mRNA and protein activity has been detected in glial cells [49]. It is tempting to speculate that RAC1 overexpression in autism may also be responsible for increased glial fibrillary acidic protein (GFAP) immunoreactivity detected in the brains of subjects with autism [54]. Higher levels of RAC1 in Caucasian adults with autism compared to African American adults with autism is a novel finding that requires further investigation, and it may be due to genetic or epigenetic factors.

Studies have demonstrated significantly elevated levels of secreted APP and its cleavage products, including beta amyloid (Aβ), in individuals with autism [55-57]. Our current results, particularly the increased expression of APP 120 kDa and 88 kDa in BA9 of children with autism, verify these earlier findings. Children with severe autism and aggression have been shown to express at least twice as much APP when compared with control children and four times as much APP when compared to children with mild autism [55]. APP translation is normally repressed by FMRP, and mGluR5 activation releases this repression, which may explain why APP increases are seen only in children and not adults [58,59]. In support of this, levels of mGluR5 were also elevated in the same specimens as previously reported [30,31], further buttressing the notion that activation of mGluR5 in children may be the cause of our observed increases in APP 88 and 120 kDa. Antipsychotic and antidepressant drugs can affect levels of APP [60,61]. However, this scenario is unlikely, as evaluation of the impact of these medications on APP values proved not to be meaningful. APP is present presynaptically in the active zone where it has multiple potential interaction partners, including synaptic vesicle proteins, transporters and channels, and cell adhesion molecules [62]. APP is also present postsynaptically where it has been shown to coprecipitate with N-methyl-D-aspartate receptor (NMDAR) subunits [63], and may be involved in surface trafficking of NMDARs [64,65]. Thus, altered expression of APP may result in altered NMDA signaling, which is crucial for learning and memory. In *Fmr1* KO mice, there are excess levels of secreted APP [59]. This excess of APP may result in decreased neural pruning and increased neural proliferation, helping to account for the presence of long, immature dendrites of neurons from patients with autism and FXS [59]. In *Fmr1* KO mice that have been engineered to remove one *App* allele, there is a significant reduction in expression of Aβ and multiple phenotypes of FXS, including the presence of audiogenic seizures, the ratio of immature dendritic spines to mature spines, and long-term depression, were partially or fully corrected after removal of the *App* allele [66]. While the presence of APP in this report probably reflects neuronal activity, increases in brain APP have also been correlated with increased GFAP in both Alzheimer’s disease [67] and autism [54] and these could also reflect glial activity.

STEP is a phosphatase that is implicated in multiple cellular processes. Kim *et al*. [68] have found increased tyrosine phosphatase activity in response to mGluR5 stimulation in *Fmr1* KO mice, leading to reduced phosphorylated extracellular signal-regulated kinases 1 and 2 (ERK1/2), molecules implicated in induction and maintenance of synaptic plasticity [69]. This tyrosine phosphatase is thought to be STEP, as STEP translation is initiated in response to mGluR stimulation [70]. Alternative splicing of the protein tyrosine phosphatase, nonreceptor type 5 (*PTPN5*) gene, which encodes STEP, produces four isoforms, however, only two - STEP 61 kDa and STEP 46 kDa - have phosphatase activity [71,72]. STEP 61 kDa is primarily membrane-bound and expressed in the postsynaptic density extrasynaptic sites, and the endoplasmic reticulum [73]. STEP 46 kDa is primarily cytosolic [73]. Other isoforms of STEP include STEP 66 kDa, STEP 27 kDa, and STEP 33 kDa. STEP 33 kDa is a cleavage product of STEP 61 kDa [74]. Recent investigations have implicated STEP in the etiology of neuropsychiatric disorders [74]. Carty *et al*. [75] reported significant increases in STEP 61 kDa in the anterior cingulate and dorsolateral prefrontal cortices of subjects with schizophrenia. STEP has roles in mediating NMDA and AMPA receptor endocytosis [70,76], suggesting that it has a role in mGluR5-mediated long-term depression (LTD), a phenomenon that is enhanced in *Fmr1* KO mice [77].

Basal levels of STEP are elevated in *Fmr1* KO mice [73]. Recent studies have shown that genetic reduction of STEP improves cognition, synaptic plasticity, and NMDA receptor subunit expression in mouse models of Alzheimer’s disease [78] and reduced seizures, anti-social, and anxiety-related behaviors in *Fmr1* KO mice [73]. We observed reduced expression patterns for STEP 66 kDa, 61 kDa, 33 kDa, and 27 kDa with only STEP 46 kDa showing a significant increase in BA9 of adults with autism. As there was an increase in one of the active forms of STEP and a decrease in the other active form, further experiments are needed to determine what role STEP might play in the pathology of autism. However, nonsignificant trends for increase in several isoforms of STEP were observed in the vermis of both children and adults with autism, with large effect sizes. Thus, it is possible that these increases are due to compensatory processes in response to reductions observed in FMRP. Evaluation of the impact of various confounders such as APDs, anticonvulsant drugs, and seizure disorder on STEP values did not prove to be meaningful, with the exception of an anticonvulsant effect on STEP46 kDa/β-actin in BA9. Data from Hasegawa *et al*. [79] show there is also minimal presence of STEP 46 kDa in reactive astrocytes of gerbil hippocampi and STEP 46 kDa/NSE data reflect STEP 46 kDa species in neuronal cells, which do not show any association with anticonvulsant use. However, evidence for seizure increasing STEP expression remains controversial [73,80], Thus, one cannot rule out the possibility of multiple factors modulating the levels of STEP molecules [75].

Homer 1 is a postsynaptic density (PSD) scaffolding protein that interacts with mGluR5 to anchor it in the postsynaptic membrane by linking mGluRs with inositol triphosphate, Shank, and PSD-95/Guanylate kinase associated protein (GKAP) [81,82]. Giuffrida *et al*. [83] found that in the postsynaptic membrane of neurons from *Fmr1* KO mice there was a reduction of mGluR5 in the insoluble fraction of the membrane as well as a reduction in phosphorylated homer 1. The interaction between homer 1 and mGluR5 regulates the cell surface expression, lateral mobility, and signaling of mGluRs [84-87]. Moreover, disruption of homer 1-mGluR5 interactions blocks mGluR5-induced long-term depression and protein synthesis in wild-type animals but not in *Fmr1* KO mice [87]. The authors suggest that in *Fmr1* KO mice, interactions between homer 1 and mGluR5 are sufficiently reduced for homer 1 to no longer have a role in the regulation of synaptic plasticity [87]. The observed reduction in homer 1 expression in BA9 of adults with autism may have functional consequences resulting in altered glutamatergic expression and cognitive deficits associated with both FXS and autism. A recent report [88] showed that homer 1 is a risk gene in autism, thus validating our novel finding. Recent evidence indicates that treatment with lithium or valproic acid causes reductions in expression of homer 1b/1c [89]. Thus, we cannot rule out a potential confounding effect of anticonvulsant drug use on homer 1/NSE in BA9 of adults, consistent with this previous finding.

Reduced FMRP expression has also been shown to cause reduction in expression of gamma aminobutyric acid (GABA) receptor subunits (α1, α3, α4, β1, β2, β3, γ1, γ2, δ) in *Fmr1* KO mice [90-93]. Moreover, in the *Drosophila melanogaster* fragile X model (*dFMR1*^−/−^), there are significant reductions in three receptors that have a high homology with ionotropic GABA receptors: resistant to dieldrin (*Rdl*), GABA and glycine-like receptor of *Drosophila* (*Grd*), and ligand-gated chloride channel homolog 3 (*Lcch3*) [91]. Our laboratory has found significantly reduced protein expression of various GABA(A) and GABA(B) receptor subunits in three brain regions (namely, the cerebellum, parietal cortex and BA9) of subjects with autism [32-34]. In BA9 we observed significantly reduced expression of GABA_A_ receptor subunit α1 (GABRα1), GABA_A_ receptor subunit α4 (GABRα4), GABA_A_ receptor subunit α5 (GABRα5), GABA_A_ receptor subunit β1 (GABRβ1), and GABA_B_ receptor subunit 1 (GABBR1) [32-34]. In the parietal cortex we observed significantly reduced expression of GABRα1, GABA_A_ receptor subunit α2 (GABRα2), GABA_A_ receptor subunit α3 (GABRα3), GABA_A_ receptor subunit β3 (GABRβ3) and GABBR1 [32-34]. In the cerebellum we observed significant reduction in expression of GABRα1, GABRβ3, GABBR1 and GABA_B_ receptor subunit 2 (GABBR2) [32-34]. More recently, using a different set of tissue samples, we observed reduced expression of GABRβ3 in the cerebellar vermis of adults with autism [31]. Our consistent findings of reduced GABA receptor protein expression in multiple brain regions of people with autism can be explained by the reduced expression of FMRP.

A further connection to this pathway includes the reduction in Reelin protein in sera and blood from individuals with autism [94,95]. Recently, mRNA for Reelin, a glycoprotein with multiple roles during development (namely, neuronal cell migration and brain lamination) and in adults (namely, modulating synaptic function), has been identified as a target of FMRP [20]. Reelin has long been considered a potential biomarker for autism due to its roles during development. Our laboratory has demonstrated reduced Reelin protein expression in sera, BA9, and the cerebella of adults with autism [94-96], supporting the idea that Reelin deficits contribute to the pathology of autism. This reduction in Reelin expression may be due to dysregulation of FMRP expression in people with autism.

**Figure 5:**
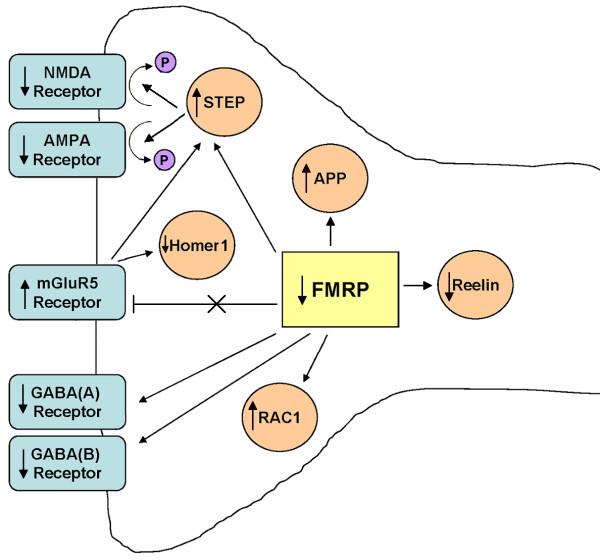
**Reduction of FMRP expression has multiple consequences.** In the absence or with reductions of FMRP, mGluR5-mediated signaling and protein synthesis is accelerated, resulting in increased expression of RAC1, APP, and STEP. Increased RAC1 and APP results in altered dendritic morphology, including presence of increased dendritic spines with a long, immature appearance. Increased STEP activity dephosphorylates multiple targets, including AMPA receptors and NMDA receptors, resulting in receptor internalization and altered synaptic transmission. Reduced homer 1 expression, reduces homer 1-mGluR5 interactions, facilitating long term depression. Reduced FMRP also results in reduced expression of GABA_A_ and GABA_B_ receptor subunits, resulting in impaired GABAergic signaling. Reduced FMRP expression may also impact Reelin expression causing further effects on synaptic transmission. The overall effect of these changes is impaired synaptic transmission and ultimately the cognitive and behavioral deficits associated with autism. FMRP, fragile X mental retardation protein; mGluR5, metabotropic glutamate receptor 5; RAC1, Ras-related C3 botulinum toxin substrate 1; amyloid beta A4 precursor protein; STEP, striatal-enriched protein tyrosine phosphatase; AMPA, 2-amino-3-(5-methyl-3-oxo-1,2- oxazol-4-yl)propanoic acid; NDMA, N-methyl-D-aspartate; GABA, gamma aminobutyric acid.

Building upon the mGluR5 theory of FXS [26] and our own findings using postmortem brain tissue from people with autism, we hypothesize that activation of mGluR5 in the brains of children with autism [30,31,97] leads to subsequent translocation of protein for FMRP from neuronal cell bodies to dendrites, and eventual decrease in FMRP at synapses [25]. Alternatively, an unknown mechanism of reduced expression of FMRP leads to increased mGluR5 signaling. These events may also lead to impaired GABAergic and Reelin signaling, ultimately leading to the morphological, cognitive, and behavioral deficits associated with autism (Figure 5). Deficits in the inhibitory GABAergic system could cause a perturbation in the excitatory/inhibitory balance in brain circuitry, potentially leading to the presence of seizures as well as impaired cognitive function. Deficits in Reelin function would also lead to abnormal brain development and abnormal synaptic transmission. Further evidence for impaired synaptic plasticity comes from altered expression of other targets of FMRP and mGluR5 signaling (namely APP, RAC1, and STEP), resulting in altered dendritic spine morphology and function as well as altered AMPA and NMDA receptor subunit composition (Figure 5). Taken together, abnormal FMRP and mGluR5 signaling in people with autism who do not have a diagnosis of FXS could explain the multiple pathologies of autism. We performed statistical correlations between levels of FMRP and mGluR5 previously reported [30,31] against RAC1, APP, STEP, and homer 1, which did not result in any significant values (data not shown). However, as relationships between these proteins are neither simple, biochemically well understood, nor stoichiometrically in a 1:1 ratio, this absence of correlation does not minimize the significance of findings in this report. Moreover, as recently shown by Darnell and Klann [98], FMRP target levels may appear unchanged or even decreased due to factors such as age, brain site, increased protein turnover and protein activation state, potentially offsetting target protein increases.

Our results also may open new ways of treating people with autism. Drugs that target metabotropic glutamate receptors have been shown to reverse morphological and behavioral deficits in *Fmr1* KO mice. AFQ056, 2-methyl-6-(phenylethynyl)-pyridine (MPEP), and 2-chloro-4-((2,5-dimethyl-1-(4-(trifluoromethoxy)phenyl)-1H-imidazol-4-yl)ethynyl)pyridine (CTEP), antagonists of mGluR5, have been shown to reduce the number of dendritic protrusions of cultured hippocampal neurons and primary visual cortex neurons from *Fmr1* KO mice [99-101]. In *Fmr1* KO mice, treatment with MPEP or AFQ056 rescues deficits in prepulse inhibition (PPI) [99,100], a sensorimotor gating behavior that is disrupted in subjects with autism, schizophrenia, and other psychiatric disorders. CTEP, a newer anti-mGluR5 inhibitor that is more potent and long lasting than MPEP, has been shown to correct for audiogenic seizures, hippocampal long-term depression, and cognitive deficits in *Fmr*1 KO mice [101]. The application of Rac1 inhibitor NSC23766 to hippocampal slices from *Fmr1* KO mice reduced long-term depression, suggesting that modulation of RAC1 could ameliorate synaptic transmission deficits associated with FXS [49]. Minocycline, a tetracycline derivative, inhibits Aβ-induced neuronal cell death, Aβ fibril formation, and microglial activation [102,103]. Minocycline can also reverse FXS phenotypes, including dendritic spine immaturity [104]. The latter therapies represent more targeted approaches, and as there are currently no approved glutamatergic inhibitors for use in humans, treatments that focus on mGluR5, RAC1 or APP may provide safe, effective means of ameliorating the behavioral deficits of autism.

## Conclusions

Taken together, the results of current postmortem studies provide novel evidence that targets of FMRP and mGluR5 signaling (RAC1, homer 1, APP, and STEP) display altered expression in the cerebellar vermis and BA9 of people with autism. There was a significant confounding effect of anticonvulant drugs on STEP 46 kDa/β-actin and a potential effect on homer 1/NSE in BA9 of adults with autism. These results are consistent with results from FXS animal models, despite the fact that none of the individuals included in the current study were diagnosed with FXS. The altered expression of proteins that may contribute to altered dendritic protein translation (homer 1), altered dendritic morphology (APP and RAC1), and receptor subunit expression (STEP), potentially contribute to the cognitive and behavioral impairments associated with autism and FXS. Our data tie in with our previous experiments, in which we have found reductions in protein expression for Reelin and GABA receptor subunit expression in multiple brain regions of people with autism. We hypothesize that reduction in FMRP and increase in mGluR5 may contribute to the dysregulation of these proteins in subjects with autism, resulting in multiple brain structural and behavioral deficits.

## Abbreviations

Aβ: Beta amyloid; AMPA: 2-Amino-3-(5-methyl-3-oxo-1,2- oxazol-4-yl)propanoic acid; APD: Antipsychotic drug; APP: Amyloid beta A4 precursor protein; BA9: Brodmann area 9; CDC: Centers for disease control and prevention; CTEP: 2-Chloro-4-((2,5-dimethyl-1-(4-(trifluoromethoxy)phenyl)-1H-imidazol-4-yl)ethynyl)pyridine; CYFIP: Cytoplasmic FMRP interacting proteins; ERK: Extracellular signal-regulated kinases; FMR1: Fragile X mental retardation 1; FRMP: Fragile X mental retardation protein; FXS: Fragile X syndrome; GABA: Gamma aminobutyric acid; GABRα: GABA_A_ receptor subunit alpha; GABRβ: GABA_A_ receptor subunit beta; GFAP: Glial fibrillary acidic protein; GKAP: Guanylate kinase associated protein; GRD: Glycine-like receptor of *Drosophila*; GTP: Guanine triphosphate; KO: Knockout; LCCH3: Ligand-gated chloride channel homolog 3; LTD: Long-term depression; mGluR5: Metabotropic glutamate receptor 5; MPEP: 2-Methyl-6-(phenylethynyl)-pyridine; NMDA: N-methyl-D-aspartate; NSE: Neuronal specific enolase; PBS: Phosphate-buffered saline; PMI: Postmortem interval; PSD: Postsynaptic density; RAC1: Ras-related C3 botulinum toxin substrate 1; RDL: Resistant to dieldrin; RT: Room temperature; STEP: Striatal-enriched protein tyrosine phosphatase; TCI: Transitory cognitive impairment.

## Competing interests

The authors declare that they have no competing interests. SH Fatemi has several patents on the use of Reelin as a diagnostic marker for neuropsychiatric disorders but has not derived any financial gains from these patents.

## Authors’ contributions

SHF conceived the study, participated in its design, supervised the conduct of all experiments, and contributed to the drafting of the manuscript and its revisions. SBL, REK, and MKH performed western blotting experiments. TDF performed western blotting experiments and contributed to the drafting of the manuscript. PDT contributed to statistical analyses of data. All authors read and approved the final manuscript.

## Supplementary Material

Additional file 1: Table S1 Western blotting results for RAC1, homer 1, APP, STEP, NSE, and β-actin and their ratios in the cerebellar vermis: controls versus people with autism not on medications^a^ (anticonvulsant, antidepressant, and antipsychotic drugs). RAC1, Ras-related C3 botulinum toxin substrate 1; APP, amyloid beta A4 precursor protein; STEP, striatal-enriched protein tyrosine phosphatase; NSE, neuronal specific enolase. Click here for file

Additional file 2: Table S2 Western blotting results for RAC1, homer 1, APP, STEP, NSE, and β-actin and their ratios in BA9: controls versus people with autism not on medications^a^ (anticonvulsant, antidepressant, and antidepressant drugs). RAC1, Ras-related C3 botulinum toxin substrate 1; APP, amyloid beta A4 precursor protein; STEP, striatal-enriched protein tyrosine phosphatase; NSE, neuronal specific enolase; BA9, Brodmann Area 9. Click here for file
