# Supplementary material for: Impairment of fragile X mental retardation protein-metabotropic glutamate receptor 5 signaling and its downstream cognates ras-related C3 botulinum toxin substrate 1, amyloid beta A4 precursor protein, striatal-enriched protein tyrosine phosphatase, and homer 1, in autism: a postmortem study in cerebellar vermis and superior frontal cortex
Source: Mol Autism. 2013 Jun 26;4:21. doi: 10.1186/2040-2392-4-21 (PMC3702477; doi:10.1186/2040-2392-4-21)
Supplement: Additional file 1: Table S1 — Western blotting results for RAC1, homer 1, APP, STEP, NSE, and β-actin and their ratios in the cerebellar vermis: controls versus people with autism not on medicationsa (anticonvulsant, antidepressant, and antipsychotic drugs). RAC1, Ras-related C3 botulinum toxin substrate 1; APP, amyloid beta A4 precursor protein; STEP, striatal-enriched protein tyrosine phosphatase; NSE, neuronal specific enolase. [file 2040-2392-4-21-S1.doc]

**Additional file 1:Table S1.** **Western blotting results for RAC1, homer 1, APP,**

**STEP, NSE, and β-actin and their ratios in cerebellar vermis: controls vs.**

**people with autism not on medications^a^ (anticonvulsants, antidepressants,**

antipsychotics)

| Adults | Control | Autistic | *P* value |
| --- | --- | --- | --- |
| RAC1/β-actin | 0.315 ± 0.206 | 1.547 ± 0.43 | 0.0038^b^ |
| Homer/β-actin | 0.237 ± 0.303 | 0.195 ± 0.23 | X |
| APP 120 kDa/β-actin | 0.091 ± 0.029 | 0.059 ± 0.02 | ns |
| APP 88 kDa/β-actin | 0.08 ± 0.06 | 0.031 ± 0.01 | X |
| STEP 66 kDa/β-actin | 0.136 ± 0.116 | 0.041 ± 0.034 | ns |
| STEP 61 kDa/β-actin | 0.015 ± 0.028 | 0.004 ± 0.006 | ns |
| STEP 46 kDa/β-actin | 0.03 ± 0.034 | 0.007 ± 0.010 | ns |
| STEP 33 kDa/β-actin | 0.55 ± 0.38 | 0.185 ± 0.160 | ns |
| STEP 27 kDa/β-actin | 0.70 ± 0.67 | 0.602 ± 0.531 | ns |
| β-actin | 10.2 ± 1.67 | 8.21 ± 0.77 | ns |
| Children | Control | Autistic | *P* value |
| RAC1/β-actin | 1.09 ± 0.545 | 1.20 ± 0.45 | ns |
| Homer/β-actin | 0.188 ± 0.13 | 0.19 ± 0.10 | ns |
| APP 120 kDa/β-actin | 0.071 ± 0.029 | 0.07 ± 0.03 | ns |
| APP 88 kDa/β-actin | 0.084 ± 0.55 | 0.09 ± 0.08 | ns |
| STEP 66 kDa/β-actin | 0.01 ± 0.008 | 0.067 ± 0.108 | ns |
| STEP 61 kDa/β-actin | 0.002 ± 0.002 | 0.034 ± 0.036 | ns |
| STEP 46 kDa/β-actin | 0.0022 ± 0.0011 | 0.026 ± 0.029 | ns |
| STEP 33 kDa/β-actin | 0.057 ± 0.085 | 0.177 ± 0.208 | ns |
| STEP 27 kDa/β-actin | 0.70 ± 0.68 | 0.645 ± 0.522 | ns |
| β-actin | 9.48 ± 1.11 | 7.73 ± 0.79 | ns |
| Adults | Control | Autistic | *P* value |
| RAC1/NSE | 0.23 ± 0.16 | 1.20 ± 0.48 | 0.011^b^ |
| Homer/NSE | 0.18 ± 0.24 | 0.16 ± 0.19 | X |
| APP 120 kDa/NSE | 0.062 ± 0.021 | 0.04 ± 0.01 | X |
| APP 88 kDa/NSE | 0.06 ± 0.05 | 0.02 ± 0.01 | X |
| STEP 66 kDa/NSE | 0.18 ± 0.14 | 0.051 ± 0.042 | ns |
| STEP 61 kDa/NSE | 0.018 ± 0.035 | 0.005 ± 0.006 | ns |
| STEP 46 kDa/NSE | 0.039 ± 0.041 | 0.029 ± 0.012 | ns |
| STEP 33 kDa/NSE | 0.72 ± 0.49 | 0.244 ± 0.202 | ns |
| STEP 27 kDa/NSE | 1.27 ± 0.82 | 0.433 ± 0.360 | X |
| NSE | 7.42 ± 1.26 | 8.17 ± 1.80 | ns |
| Children | Control | Autistic | *P* value |
| RAC1/NSE | 0.84 ± 0.51 | 0.78 ± 0.17 | ns |
| Homer/NSE | 0.13 ± 0.09 | 0.15 ± 0.07 | ns |
| APP 120 kDa/NSE | 0.040 ± 0.025 | 0.05 ± 0.02 | ns |
| APP 88 kDa/NSE | 0.054 ± 0.025 | 0.07 ± 0.06 | ns |
| STEP 66 kDa/NSE | 0.014 ± 0.011 | 0.121 ± 0.199 | ns |
| STEP 61 kDa/NSE | 0.0024 ± 0.001 | 0.056 ± 0.062 | ns |
| STEP 46 kDa/NSE | 0.003 ± 0.002 | 0.046 ± 0.055 | ns |
| STEP 33 kDa/NSE | 0.078 ± 0.12 | 0.320 ± 0.389 | ns |
| STEP 27 kDa/NSE | 0.36 ± 0.24 | 1.107 ± 1.000 | ns |
| NSE | 6.97 ± 0.84 | 5.48 ± 1.41 | ns |

^a^RAC1, ras-related C3 botulinum toxin substrate 1; APP, amyloid beta A4

precursor protein; ns, not significant; ^b^statistically significant; X, t-test can not be

performed due to low n.; ns, not significant
