# Supplementary material for: Impairment of fragile X mental retardation protein-metabotropic glutamate receptor 5 signaling and its downstream cognates ras-related C3 botulinum toxin substrate 1, amyloid beta A4 precursor protein, striatal-enriched protein tyrosine phosphatase, and homer 1, in autism: a postmortem study in cerebellar vermis and superior frontal cortex
Source: Mol Autism. 2013 Jun 26;4:21. doi: 10.1186/2040-2392-4-21 (PMC3702477; doi:10.1186/2040-2392-4-21)
Supplement: Additional file 2: Table S2 — Western blotting results for RAC1, homer 1, APP, STEP, NSE, and β-actin and their ratios in BA9: controls versus people with autism not on medicationsa (anticonvulsant, antidepressant, and antidepressant drugs). RAC1, Ras-related C3 botulinum toxin substrate 1; APP, amyloid beta A4 precursor protein; STEP, striatal-enriched protein tyrosine phosphatase; NSE, neuronal specific enolase; BA9, Brodmann Area 9. [file 2040-2392-4-21-S2.doc]

**Additional file 2: Table S2. Western blotting results for RAC1, homer 1, APP,**

**STEP, NSE, and β-actin and their ratios in BA9: controls vs. people with**

**autism not on medications^a^ (anticonvulsants, antidepressants, and**

antidepressants)

| Adults | Control | Autistic | *P* value |
| --- | --- | --- | --- |
| RAC1/β-actin | 0.834 ± 0.554 | 1.95 ± 0.59 | 0.011^b^ |
| Homer/β-actin | 0.512 ± 0.152 | 0.39 ± 0.13 | ns |
| APP 120 kDa/β-actin | 0.41 ± 0.23 | 0.51 ± 0.18 | ns |
| APP 88 kDa/β-actin | 0.46 ± 0.19 | 0.60 ± 0.19 | ns |
| STEP 66 kDa/β-actin | 0.10 ± 0.07 | 0.1 ± 0.067 | ns |
| STEP 61 kDa/β-actin | 0.73 ± 0.17 | 0.73 ± 0.19 | ns |
| STEP 46 kDa/β-actin | 0.0097 ± 0.006 | 0.02 ± 0.09 | ns |
| STEP 33 kDa/β-actin | 0.20 ± 0.13 | 0.21 ± 0.09 | ns |
| STEP 27 kDa/β-actin | 0.20 ± 0.12 | 0.18 ± 0.09 | ns |
| β-actin | 13.2 ± 2.41 | 9.53 ± 0.85 | ns |
| Children | Control | Autistic | *P* value |
| RAC1/β-actin | 1 ± 0.616 | 2.96 ± 0.68 | 0.01 |
| Homer/β-actin | 0.46 ± 0.17 | 0.83 ± 0.32 | ns |
| APP 120 kDa/β-actin | 0.20 ± 0.07 | 0.66 ± 0.28 | 0.043^b^ |
| APP 88 kDa/β-actin | 0.22 ± 0.03 | 0.82 ± 0.26 | 0.017^b^ |
| STEP 66 kDa/β-actin | 0.027 ± 0.026 | 0.048 ± 0.034 | ns |
| STEP 61 kDa/β-actin | 0.59 ± 0.22 | 0.29 ± 0.27 | ns |
| STEP 46 kDa/β-actin | 0.008 ± 0.006 | 0.01 ± 0.006 | ns |
| STEP 33 kDa/β-actin | 0.017 ± 0.026 | 0.038 ± 0.026 | ns |
| STEP 27 kDa/β-actin | 0.17 ± 0.17 | 0.26 ± 0.11 | ns |
| β-actin | 11.5 ± 1.31 | 9.26 ± 0.94 | ns |
| Adults | Control | Autistic | *P* value |
| RAC1/NSE | 0.50 ± 0.33 | 1.12 ± 0.30 | ns |
| Homer/NSE | 0.34 ± 0.12 | 0.25 ± 0.08 | ns |
| APP 120 kDa/NSE | 0.25 ± 0.12 | 0.29 ± 0.12 | ns |
| APP 88 kDa/NSE | 0.28 ± 0.07 | 0.34 ± 0.10 | ns |
| STEP 66 kDa/NSE | 0.17 ± 0.13 | 0.16 ± 0.10 | ns |
| STEP 61 kDa/NSE | 1.3 ± 0.34 | 1.12 ± 0.34 | ns |
| STEP 46 kDa/NSE | 0.017 ± 0.011 | 0.033 ± 0.032 | ns |
| STEP 33 kDa/NSE | 0.35 ± 0.20 | 0.33 ± 0.14 | ns |
| STEP 27 kDa/NSE | 0.35 ± 0.21 | 0.29 ± 0.13 | ns |
| NSE | 9.14 ± 3.73 | 7.80 ± 1.12 | ns |
| Children | Control | Autistic | *P* value |
| RAC1/NSE | 0.61 ± 0.42 | 1.32 ± 0.30 | X |
| Homer/NSE | 0.28 ± 0.11 | 0.42 ± 0.16 | ns |
| APP 120 kDa/NSE | 0.12 ± 0.04 | 0.34 ± 0.13 | 0.047^b^ |
| APP 88 kDa/NSE | 0.13 ± 0.004 | 0.41 ± 0.12 | 0.014^b^ |
| STEP 66 kDa/NSE | 0.072 ± 0.081 | 0.098 ± 0.075 | ns |
| STEP 61 kDa/NSE | 1.03 ± 0.25 | 0.62 ± 0.56 | ns |
| STEP 46 kDa/NSE | 0.021 ± 0.021 | 0.019 ± 0.014 | ns |
| STEP 33 kDa/NSE | 0.050 ± 0.083 | 0.081 ± 0.061 | ns |
| STEP 27 kDa/NSE | 0.49 ± 0.57 | 0.54 ± 0.31 | ns |
| NSE | 6.58 ± 2.48 | 6.06 ± 1.01 | ns |

^a^RAC1, ras-related C3 botulinum toxin substrate 1; APP, amyloid beta A4

precursor protein; BA9, Brodmann’s area 9; ns, not significant; ^b^statistically

significant; X, t-test can not be performed due to low n. ns, not significant
